# Osteo-Chondroprogenitor–Specific Deletion of the Selenocysteine tRNA Gene, *Trsp*, Leads to Chondronecrosis and Abnormal Skeletal Development: A Putative Model for Kashin-Beck Disease

**DOI:** 10.1371/journal.pgen.1000616

**Published:** 2009-08-21

**Authors:** Charlene M. Downey, Chelsea R. Horton, Bradley A. Carlson, Trish E. Parsons, Dolph L. Hatfield, Benedikt Hallgrímsson, Frank R. Jirik

**Affiliations:** 1 The McCaig Institute for Bone and Joint Health, University of Calgary, Calgary, Alberta, Canada; 2 Department of Biochemistry and Molecular Biology, University of Calgary, Calgary, Alberta, Canada; 3 Molecular Biology of Selenium Section, Laboratory of Cancer Prevention, Center for Cancer Research, National Cancer Institute, National Institutes of Health, Bethesda, Maryland, United States of America; 4 Department of Cell Biology and Anatomy, University of Calgary, Calgary, Alberta, Canada; University of Washington, United States of America

## Abstract

Kashin-Beck disease, a syndrome characterized by short stature, skeletal deformities, and arthropathy of multiple joints, is highly prevalent in specific regions of Asia. The disease has been postulated to result from a combination of different environmental factors, including contamination of barley by mold mycotoxins, iodine deficiency, presence of humic substances in drinking water, and, importantly, deficiency of selenium. This multifunctional trace element, in the form of selenocysteine, is essential for normal selenoprotein function, including attenuation of excessive oxidative stress, and for the control of redox-sensitive molecules involved in cell growth and differentiation. To investigate the effects of skeletal selenoprotein deficiency, a Cre recombinase transgenic mouse line was used to trigger *Trsp* gene deletions in osteo-chondroprogenitors. *Trsp* encodes selenocysteine tRNA^[Ser]Sec^, required for the incorporation of selenocysteine residues into selenoproteins. The mutant mice exhibited growth retardation, epiphyseal growth plate abnormalities, and delayed skeletal ossification, as well as marked chondronecrosis of articular, auricular, and tracheal cartilages. Phenotypically, the mice thus replicated a number of the pathological features of Kashin-Beck disease, supporting the notion that selenium deficiency is important to the development of this syndrome.

## Author Summary

Kashin-Beck disease (KBD) is a severe, chronic, and deforming musculoskeletal disease affecting millions of individuals in specific regions of Asia. Starting in childhood, the disorder leads to joint and limb deformities, short stature, and delayed skeletal development. Articular cartilage damage due to chondronecrosis and limb deformities then lead to secondary osteoarthritis and severe disability. Factors proposed to cause KBD include selenium deficiency, iodine deficiency, contamination of grain with toxic molds, and humic substances in well water. Soil and water deficiency in selenium (and iodine) are a consistent feature of KBD endemic areas, and affected individuals show profound deficiencies of these two elements. Thus far, there have been no convincing rodent models of KBD based on selenium (and/or iodine) deficiency achieved through dietary manipulation. Our manuscript describes a conditional gene mutation approach in mice that, in effect, mimics severe selenium deficiency, achieving this specifically within skeletal progenitor cells. By deleting selenocysteine tRNA (required for normal selenoprotein activity) in osteo-chondroprogenitors, we found that mice develop post-natal impairment of skeletal growth, dwarfism, delayed ossification, impaired endochondral bone formation, as well as severe chondronecrosis. Our mutant mouse supports the idea that selenium deficiency is key to the skeletal pathology of KBD.

## Introduction

Kashin-Beck disease, an environmentally-induced musculoskeletal syndrome, is prevalent in millions of individuals residing within specific regions of Tibet, China, Siberia, and North Korea [1]–[3]. The disease first becomes evident in childhood with affected individuals exhibiting short stature, joint and limb deformities, and radiographic evidence of delayed skeletal ossification [4],[5]; features attributed to impaired epiphyseal growth and chondronecrosis [1],[3],[4],[6]. With age, severe secondary osteoarthritis of multiple joints becomes evident. Although several factors have been implicated in the pathogenesis of this disease, deficiency of dietary selenium intake, and hence, profoundly low serum selenium levels represent one of the most salient features of Kashin-Beck disease [1],[7],[8]. This raised the possibility that deficiencies in one or more selenoproteins might play key etiological roles in this musculoskeletal disorder.

Selenium is an essential dietary micronutrient that is associated with various organic molecules, including the 21^st^ amino acid, selenocysteine (Sec), that is required for the function of a class of proteins known as the selenoproteins [9]. There are 25 human and 24 murine genes that encode for selenoproteins, approximately one third of which function as antioxidants, protecting cells against macromolecule damage caused by oxidative and/or nitrosative stress [10]. Other members of the selenoprotein family include the deiodinases, including, for example, deiodinase 2 (D2) that catalyzes conversion of thyroxine (T_4_) into the active tri-iodothyronine (T_3_) form; the latter has many roles in growth and development, including the regulation of epiphyseal growth plate differentiation [11]–[13]. Selenoprotein synthesis requires that Sec residues be inserted into the growing polypeptide chains via specific UGA codons present within the coding regions of selenoprotein mRNAs. Although UGA specifies a ‘stop’ codon in the universal genetic code, it can also code for Sec with the participation of a group of proteins that recognize the Sec insertion sequence (SECIS) element located in the 3′ untranslated region of selenoprotein mRNAs [14]. UGA is recognized by Sec tRNA (designated Sec tRNA^[Ser]Sec^) which is encoded by *Trsp* (present in one copy per haploid genome).

Sec tRNA^[Ser]Sec^ is responsible for the expression of all selenoproteins and thus it provides a unique tool for studying the role of selenium within this class of proteins. The targeted deletion of *Trsp* is embryonic lethal owing to a loss of activity of selenoproteins that are essential for normal growth and development [15]. Hence, investigation of the tissue-specific consequences of selenoprotein loss *in vivo* must be carried out via a conditional mutagenesis approach. *Cre*-*lox*P technology has been successfully used to study the consequences of floxed *Trsp* gene *(Trsp^fl/fl^*) excision in cardiac muscle, hepatocytes, lymphocytes, mammary epithelium, and neurons, with pathological changes attributable to selenoprotein deficiency being demonstrated in all these tissues [9].

In addition to dietary deficiency of selenium, deficiency of iodine, ingestion of fungal mycotoxins from contaminated stored food, high humic acid levels in the drinking water, or some combination of these components have been proposed to have roles in the genesis of Kashin-Beck disease [1],[7],[8],[16]. The relative contributions of the various environmental factors implicated in Kashin-Beck disease are not known but an emphasis has been placed on the role of selenium deficiency [1],[17],[18]. Induction of dietary selenium and/or iodine deficiency in rodents, however, fails to replicate many of the clinical features of Kashin-Beck disease, while showing only modest effects on skeletal growth and bone volume [17],[18]. This could be due to the difficulty in attaining the levels of selenium deficiency observed in Tibetan children which are below 27 ng/ml in 90% of affected individuals. Values in the 5 ng/ml range are observed in a third of affected individuals [1]. In contrast, rats maintained on selenium deficient diets only exhibited serum levels in the 30 ng/ml range [17].

We hypothesized that interfering with cartilage selenoprotein synthesis, and hence mimicking the effects of severe selenium deprivation in this tissue, might recapitulate features of Kashin-Beck disease, including delayed endochondral bone development, impaired ossification, chondronecrosis, and dwarfism. To test this hypothesis, we deleted the *Trsp* gene in cells that give rise to the skeleton. This was accomplished by generating *Trsp^fl/fl^* mice expressing the Cre recombinase under the control of the *Col2a1* gene promoter in order to obtain *Trsp* deletions in osteo-chondroprogenitors [19],[20].

## Results/Discussion

### Targeted deletion of *Trsp* in skeletal precursor cells leads to impaired skeletal growth and development and premature death

Previous studies have shown that the generalized knockout of *Trsp* is lethal at the embryonic stage [15],[21]. To produce viable individuals, we crossed a mouse with floxed *Trsp* alleles with a transgenic line expressing the Cre recombinase under the control of the *Col2a1* promoter. *Col2a1-Cre* mice exhibited Cre-mediated gene excision activity starting at approximately 9 days post coitum (dpc) in notochord and cranial mesenchyme (sites where the endogenous Col2a1 gene begins to be expressed), with Cre reporter activity being evident in all cartilage primordia (ribs, long bones, spine, basicranium) by 15 dpc [19]. Furthermore, using the same β-galactosidase-based Cre reporter strain as Ovchinnikov et al. [19], we detected Cre activity not only in mature cartilage and endochondrally derived bone, but also within regions of the cranium resulting from intra-membranous ossification [20]. These results suggested that osteo-chondroprogenitors were the targets for Cre-mediated genomic alterations in *Col2a1-Cre* transgenic mice.

**Figure 1:**
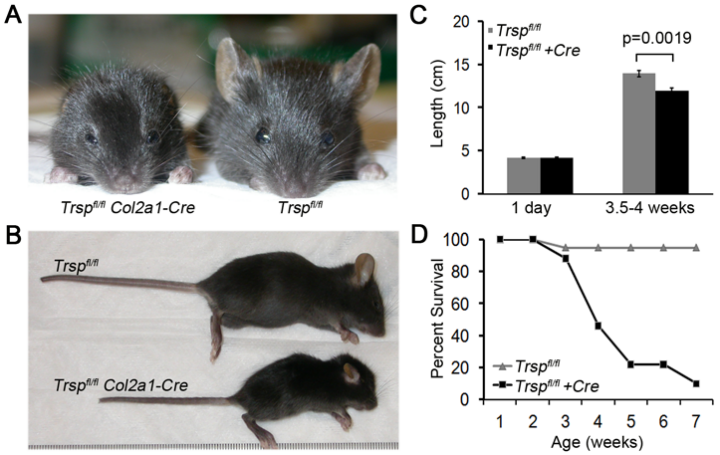
Trsp^fl/fl^ Col2a1-Cre mice exhibit runting and a shortened life-span. Frontal (A) and lateral (B) views of representative 4 wk old *Trsp^fl/fl^ Col2a1-Cre* and control mice. Compared to the littermate control, the *Trsp^fl/fl^ Col2a1-Cre* animal is runted, with hypomorphic ears and a shortened snout. (C) Lengths (from nose tip to base of tail) of groups of mice taken either at 1 day of age (n = 7 *Trsp^fl/fl^ Col2a1-Cre*, n = 4 *Trsp^fl/fl^*), or at between 3.5–4 weeks of age (n = 9 *Trsp^fl/fl^ Col2a1-Cre*, n = 8 *Trsp^fl/fl^*). While lengths of control and experimental mice were similar in the immediate post-natal period, by 3.5–4 weeks their lengths had diverged significantly. Data are mean+/−SEM. (D) Survival rates of mice followed for up to 7 weeks of age (n = 17 *Trsp^fl/fl^ Col2a1-Cre*; n = 18 *Trsp^fl/fl^*).

Compared to littermate controls, *Col2a1-Cre*; *Trsp^fl/fl^* mice demonstrated marked reduction in skeletal and cartilage growth. As early as 1 wk after birth, the mutant mice began to exhibit dwarfism, marked auricular hypoplasia, shortened snouts, decreased head size with frontal bossing, smaller limbs and shorter tails (Figure 1A and 1B). Males and females were equally affected. Interestingly, *Col2a1-Cre*; *Trsp^fl/fl^* mice were indistinguishable from control mice within the first few days after birth. Indeed, mouse lengths (nose to tail base) measured on post-natal day 1, showed no difference between the two groups (Figure 1C). In contrast, by 3.5–4 weeks of age, the differences in length of the two groups had become significant (Figure 1C), indicating that an additional factor was involved, such as exposure to ambient oxygen. The 3.5–4 wk time point was selected for analyses owing to the high incidence of death (or need for euthanasia) in 4–5 wk old *Col2a1-Cre*; *Trsp^fl/fl^* mice (Figure 1D). Moribund animals demonstrated marked rib cage indrawing, suggestive of inspiratory respiratory distress.

### Impaired ossification, shortening of the long bones, and widening of tibial growth plates in *Col2a1-Cre; Trsp^fl/fl^* mice

**Figure 2:**
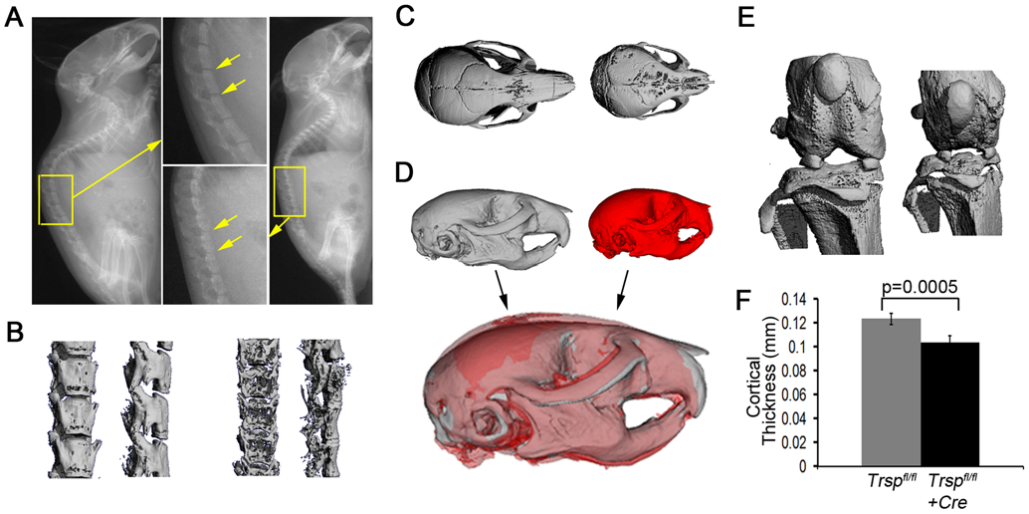
Compromised skeletal development in Trsp^fl/fl^ Col2a1-Cre mice. (A) Radiographs of representative *Trsp^fl/fl^* (left) and *Trsp^fl/fl^ Col2a1-Cre* (right) mice. Arrows within the magnified areas highlight the irregularly-shaped vertebral bodies and narrowed intervertebral disc spaces in the *Trsp^fl/fl^ Col2a1-Cre* spinal column. (B) Ventral and lateral micro-CT views of lumbar vertebrae from *Trsp^fl/fl^* control (left) and *Trsp^fl/fl^ Col2a1-Cre* (right) mice showing delayed spinal column ossification and loss of intervertebral disc spaces. (C) Micro-CT scans of the dorsal aspect of control (left) and experimental (right) skulls show smaller overall head size, rounding of the cranium (with possible decreased frontal bone ossification), and nasal bone shortening in the *Trsp^fl/fl^ Col2a1-Cre* mouse. (D) Generalized shape images of representative skulls. Ten mutant mice were used to generate this phenotypic average for the *Trsp^fl/fl^ Col2a1-Cre* mice (red skull) and nine *Trsp^fl/fl^* controls were used (grey skull); the overlay of the images is presented beneath. Note that skull sizes from the control and experimental groups have been normalized, thus, only differences in shape of the cranium are being revealed by the overlay technique. (E) Anterior micro-CT views of knee joints from control (left) and experimental (right) mice showing smaller overall size in the latter, yet with relatively wide growth plates being present in the *Trsp^fl/fl^ Col2a1-Cre* tibia and fibula. (F) Cortical bone thickness difference (p = 0.0005) between the genotypes at the mid-shaft of the femur measured by micro-CT (n = 5). Data are mean+/−SEM, and were from 3.5–4 wk old mice.

Radiographs of littermate control and *Col2a1-Cre*; *Trsp^fl/fl^* mice (Figure 2A) demonstrated the smaller skeletons of the mutant mice, and the rounding of the cranium. The spaces between the vertebral bodies, due to the intervertebral discs, were narrowed in the mutant mice as compared with the littermate controls (Figure 2A). The lumbar vertebrae of the *Col2a1-Cre*; *Trsp^fl/fl^* mice were smaller, with irregular outlines and were unevenly ossified. These findings were confirmed by microcomputed tomography (micro-CT) imaging of the lumbar vertebrae (Figure 2B). Micro-CT also demonstrated the reduced cranial size and abnormal cranial shape in *Col2a1-Cre*; *Trsp^fl/fl^* mice (Figure 2C and 2D), with snout shortening, narrowing of the jaw, and rounding of the calvaria. This pattern is consistent with reduction in the growth of the chondrocranial component of the skull. Indeed, other mutations that perturb chondrocranial growth exhibit similar, albeit less extreme, effects on the shape of the mouse skull [22],[23]. The more porous nature of the frontal bones in the mutant, evident in the micro-CT image of the cranium (Figure 2C), may have been reflective of impaired intramembranous bone development secondary to *Col2a1*-*Cre* mediated deletions of *Trsp* in osteoblasts [20].

**Figure 3:**
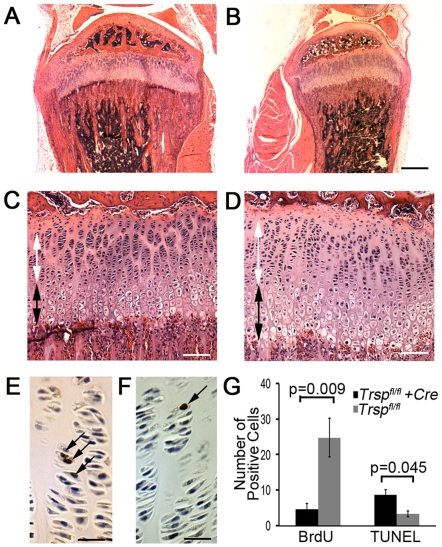
Tibial epiphyseal growth plate abnormalities in Trsp^fl/fl^ Col2a1-Cre mice. Relative sizes and morphological differences between the tibiae of (A) *Trsp^fl/fl^* (control), and (B) *Trsp^fl/fl^ Col2a1-Cre* mice (hematoxylin and eosin stained sections) are shown. Note smaller bone size, and the disproportionate widening of the epiphyseal growth plate in the *Trsp^fl/fl^ Col2a1-Cre* mice. Higher magnification views of representative growth plate sections from *Trsp^fl/fl^* (C) and *Trsp^fl/fl^ Col2a1-Cre* (D) mice; there was widening of both the proliferative (white two-headed arrows) and hypertrophic (black two-headed arrows) zones in the mutant mice. BrdU+ nuclear staining revealed an increase in the number of mitoses within the tibial growth plate proliferative zones of *Trsp^fl/fl^* (E) as compared to *Trsp^fl/fl^ Col2a1-Cre* (F) mice (arrows indicate examples of BrdU+ cells). Scale bars were as follows: 450 µm (A and B), 55 µm (C and D) and 27 µm (E and F). (G) Numbers of BrdU+ nuclei (n = 14 *Trsp^fl/fl^ Col2a1-Cre* and n = 7 *Trsp^fl/fl^* tibiae), and TUNEL positive apoptotic cells (n = 3 *Trsp^fl/fl^ Col2a1-Cre* and n = 5 *Trsp^fl/fl^* tibiae) present in the epiphyseal growth plates. Data are shown as mean+/−SEM.

Micro-CT confirmed that smaller knees were a feature of the mutant mice (Figure 2E). Quantitative micro-CT of femoral cortical bone demonstrated that the *Col2a1-Cre*; *Trsp^fl/fl^* long bones were impaired in their mineralization (p = 0.005) when compared to the bones of littermate controls (Figure 2F). Interestingly, despite the reduced size of the knees, the width of the radiolucent gap corresponding to the tibial epiphyseal growth plates in was not correspondingly reduced in the mutant mice. Histological sections of mutant mice tibiae revealed increased epiphyseal growth plate width despite reduced bone length. The epiphyses were also characterized by a disorganized primary spongiosa in comparison to littermate controls (Figure 3A and 3B). Indeed, there was an approximate 45% increase in tibial growth plate width in the mutant mice (data not shown) that was due to increases in both the proliferative and hypertrophic chondrocyte zones (Figure 3C and 3D). This increased width was present despite the finding of decreased cell proliferation (as shown by 5-bromo-2-deoxyuridine, BrdU, incorporation) and only modest increases in TUNEL (terminal deoxynucleotidyl transferase dUTP nick end-labeling) positive apoptotic cells in the mutant growth plates (Figure 3E–3G). We speculate that accumulation of cells in the proliferative zone may have been the result of a slowing of the hypertrophic differentiation process, and that hypertrophic zone expansion may have been due to impaired osteoblastic invasion of this layer.

### Abnormalities of cartilage in *Col2a1-Cre; Trsp^fl/fl^* mice

**Figure 4:**
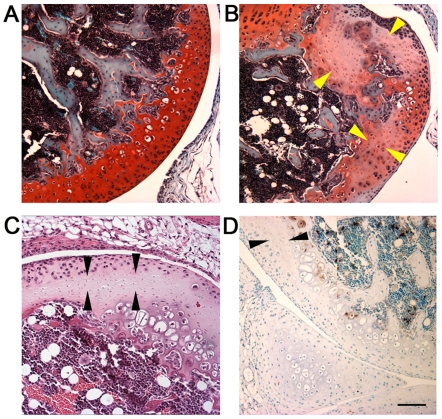
Chondronecrosis in the articular cartilage of Trsp^fl/fl^ Col2a1-Cre mice. (A) Triple-stained section of the femoral articular cartilage of a *Trsp^fl/fl^* control mouse; the red staining reveals the presence of glycosaminoglycans in the extracellular matrix surrounding the embedded chondrocytes. (B) Triple-stain of femoral articular cartilage from a representative *Trsp^fl/fl^ Col2a1-Cre* mouse, showing marked glycosaminoglycan depletion (pale red regions) in the areas of chondronecrosis (arrowheads). (C) Hematoxylin and eosin-stained section of distal femoral articular cartilage from another *Trsp^fl/fl^ Col2a1-Cre* mouse, showing a typical region of chondronecrosis (arrowheads). (D) TUNEL positive apoptotic cells (stained brown) lining an area of chondronecrosis (arrowheads) in the distal femoral articular cartilage of a *Trsp^fl/fl^ Col2a1-Cre* mouse knee. Scale bar = 110 µm.

**Figure 5:**
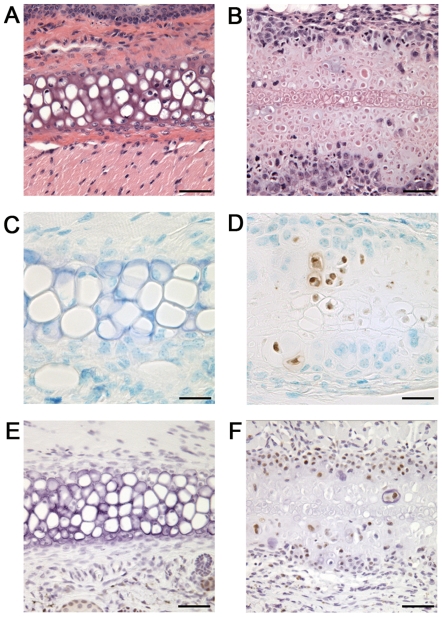
Auricular chondronecrosis and hypoplasia in Trsp^fl/fl^ Col2a1-Cre mice. (A) Hematoxylin and eosin staining of ear cartilage from a control *Trsp^fl/fl^* mouse, compared with (B) *Trsp^fl/fl^ Col2a1-Cre* auricular cartilage that shows a wide band of necrotic chondrocytes. Detection of TUNEL positive cells (stained brown, with methyl-green counterstain) in *Trsp^fl/fl^* control (C) and *Trsp^fl/fl^ Col2a1-Cre* (D) auricular cartilage. Note abundance of necrotic cells with scattered TUNEL positive cells in (D). Evidence of apparent regenerative activity of cartilage above and below the band of necrotic cartilage, but not in the control (E), is shown (F). Detection of cell proliferation using anti-PCNA antibody immunostaining on control *Trsp^fl/fl^* (E) and *Trsp^fl/fl^ Col2a1-Cre* (F) cartilage; haematoxylin counter stain. Scale bars are as follows: 55 µm (A,B,E,F), 27 µm (C,D).

**Figure 6:**
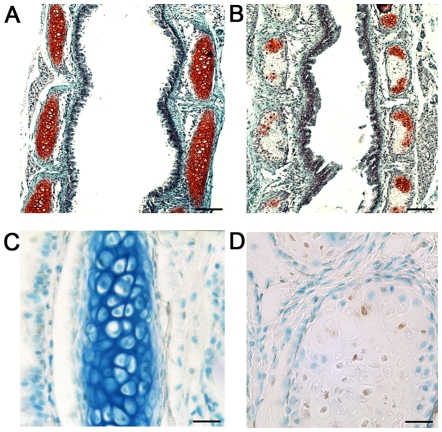
Tracheomalacia and airway lumen narrowing in Trsp^fl/fl^ Col2a1-Cre mice. (A) Triple-stained section of a trachea from a representative *Trsp^fl/fl^* (control) mouse, showing normal cartilage ring cross-sections with abundant cells and glycosaminoglycan deposition in the extracellular matrix (indicated by red staining); the connective tissue is stained green. (B) Tracheal rings from a representative *Trsp^fl/fl^ Col2a1-Cre* mouse were reduced in size, and showed a dramatic loss of red staining as well as chondrocyte depletion; dystrophic calcification was also present in some cartilage rings, for example, the dark staining region in uppermost ring on the right side of the trachea in (B). The lumen of the *Trsp^fl/fl^ Col2a1-Cre* tracheas was also narrowed. TUNEL positive cells were not evident in the control tracheal cartilages (C), however, scattered TUNEL positive chondrocytes were present within areas of chondronecrosis within *Trsp^fl/fl^ Col2a1-Cre* tracheal rings (D). Methyl-green counterstaining, used to indicate positions of cell nuclei, also revealed loss of extracellular matrix staining in the *Trsp^fl/fl^ Col2a1-Cre* rings (D) consistent with chondrocyte dysfunction or death. Scale bars are 450 µm (A and B), and 27 µm (C and D).

Areas of chondronecrosis, a key pathological feature of Kashin-Beck disease [3],[4],[6] were observed in cartilaginous tissues, including the articular cartilage, ears, and tracheal rings. The articular cartilage of a littermate control knee shown in Figure 4A exemplifies the way that cartilage tissue sections should appear when stained with hematoxylin, fast green and safranin O, with the red stain indicating the presence of the glycosaminoglycans. In contrast, areas of chondronecrosis were observed in the articular cartilage of *Col2a1-Cre*; *Trsp^fl/fl^* knees (Figure 4B and 4C), as indicated through the loss of chondrocytes and glycosaminoglycan staining. These areas of chondronecrosis were flanked by sparse TUNEL positive apoptotic cells (Figure 4D). Chondronecrosis was confined primarily to the femoral condylar and tibial plateau articular cartilage, but were not evident within the tibial epiphyseal growth plates. Massive chondronecrosis was evident in the hypoplastic ears of the mutant mice (Figure 5A and 5B), again these areas contained small numbers of TUNEL positive chondrocytes (Figure 5C and 5D). Neutrophil infiltrates, a feature of necrotic cell death, surrounded some of the chondronecrotic areas in the ears (data not shown), and these necrotic regions were often flanked by clusters of proliferating cells (Figure 5E and 5F), possibly reflective of cartilage regeneration. Lastly, premature death in *Col2a1-Cre*; *Trsp^fl/fl^* mice appeared to result from respiratory distress, and could be explained by tracheal narrowing and/or collapse upon inspiration due to loss of tracheal ring integrity due to the tracheomalacia. In keeping with this idea, we found marked hypoplasia and chondronecrosis (Figure 6A and 6B), along with scattered TUNEL positive apoptotic cells (Figure 6C and 6D), in *Col2a1-Cre*; *Trsp^fl/fl^* tracheal cartilages. To the best of our knowledge, there are no reports of an increase frequency of tracheomalacia in newborns within Kashin-Beck disease endemic areas.

**Figure 7:**
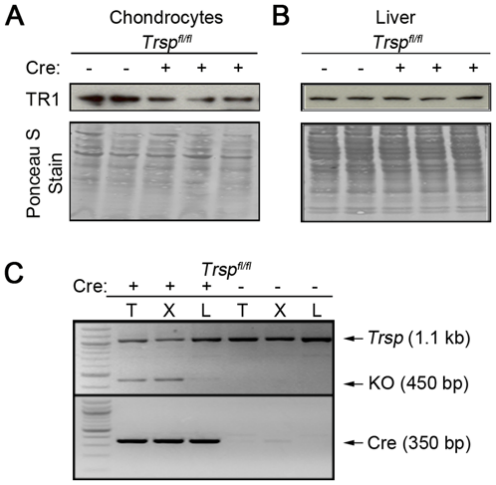
Immunoblotting analysis of TR1 in chondrocytes and demonstration of Trsp excisions in cartilage-rich tissues. Protein extracts were prepared from (A) chondrocytes and (B) liver samples from two control (*Trsp^fl/fl^*) and three *Trsp^fl/fl^ Col2a1-Cre*) mice. Lysates were then subjected to electrophoresis, transblotted, and probed with an anti-TR1 antibody (upper panels) as described in the Materials and Methods section. The lower panels show the Ponceau S-stained membranes of the total protein extracts as loading controls. Representative blots are shown. (C) Representative genotyping results from tail tip, xiphisternal and liver DNA samples from control and experimental mice. PCR reactions to detect *Trsp^fl/fl^* 450 bp excision band are shown in the upper panel; and PCR demonstrating the presence of the *Col2a1-Cre* transgene are in the lower panel.

To assess whether the targeted removal of *Trsp* in chondrocytes resulted in a down-regulation of selenoprotein expression, immunoblot analysis of chondrocytes for one of the major housekeeping selenoproteins, thioredoxin reductase (TR1), was performed (Figure 7A and 7B). This revealed a decrease in TR1 levels in the chondrocytes of knockout mice as compared to control mice. In contrast, TR1 levels were virtually unchanged in liver lysates, which served as the control tissue. The reason a complete loss in TR1 expression was not observed in the chondrocyte preparation may have been due to contaminating cell types such as fibroblasts in the lysate preparations [24], and/or to incomplete excision of floxed *Trsp* by Cre recombinase in chondrocytes [15]. Genomic PCR revealed that *Trsp* excisions were present in the cartilage-rich tissues (xiphoid process, tail tip) of *Trsp^fl/fl^ Col2a1-Cre* mice; however, the appearance of the un-excised band in these tissues was again likely due to the presence of non-chondrocytic cells in the tissue samples (Figure 7C). There was a faint excision band present in the liver DNA samples, suggesting that there was some low level of ‘leakiness’ of the Cre transgene in this tissue. In summary, *Trsp* deletions were present in the cartilage-rich tissues of the mutant mice, and this was accompanied by a decrease in the expression level of an important selenoprotein, TR1.

The chondronecrosis and impaired skeletogenesis seen in *Col2a1-Cre*; *Trsp^fl/fl^* mice potentially results from chondrocyte and osteoblast deficiencies in a range of different anti-oxidant selenoproteins, including specific glutathione peroxidases (such as glutathione peroxidases 1 and 4) and, as we have seen, thioredoxin reductases (such as TR1) [9]. The antioxidant selenoproteins constitute an important line of defense against cellular damage, necrosis, and apoptosis brought about by excessive levels of reactive oxygen and nitrogen species [25]. The thioredoxin reductases, for example, are required for normal thioredoxin function, and as such support cell proliferation and the activities of specific redox-sensitive molecules, such as specific transcription factors or their regulators [26]–[28]. The idea that the post-natal onset of runting in *Col2a1-Cre*; *Trsp^fl/fl^* mice may have been due to exposure to ambient oxygen, and the ensuing increase in oxidative stress, would be consistent with anti-oxidant defenses of chondrocytes and osteoblasts having been compromised.

It was also possible that intracellular T_3_ deficiency, resulting from decreased selenoprotein deiodinase activity, may have contributed to the phenotype of *Col2a1-Cre*; *Trsp^fl/fl^* mice. The thyroid secretes T_4_ into the circulation which must then be converted within target tissues into the active T_3_ form by intracellular D2 [29],[30]. T_3_, a hormone utilized by all tissues, is also required for normal growth plate development [11], and nuclear receptors for this ligand have been shown to be expressed in skeletal cells [31]. Although diminished D2 deiodinase activity could theoretically lead to a tissue-specific deficiency of T_3_, genetic deletion of D2, either alone or in combination with D1, yielded no evidence of impaired skeletal growth [32]. This is in keeping with recent data indicating that neither D1 or D2 are expressed in rodent chondrocytes, and that D2 is only found in mature osteoblasts [33]. In contrast, D3, a deiodinase that down-regulates intracellular T3 levels in the skeleton (to avoid accelerated bone maturation), is expressed in young rodent chondrocytes and osteoblasts. Thus, mice lacking D3 exhibited generalized growth retardation attributed to perinatal thyrotoxicosis and that was subsequently compounded by severe hypothyroidism starting around the time of weaning [34]. In view of these results, we hypothesize that osteo-chondroprogenitor specific deficiency of the D3 selenoprotein in *Col2a1-Cre*; *Trsp^fl/fl^* mice may have allowed the accumulation of abnormally high concentrations of intracellular T3 during the perinatal period. Raised T3 levels would not only act to accelerate bone maturation, but would also contribute to oxidative stress [35], thus aggravating macromolecule damage in cells already impaired in their selenoprotein-based anti-oxidant defenses.

It should be noted, however, that congenital thyroid hormone deficiency in humans has been associated with multiple skeletal effects, including short stature, vertebral and cranial abnormalities, as well as delayed endochondral bone formation and skeletal maturation [36]. These phenotypic features, which may also be due in part to neuro-endocrine axis abnormalities, are manifested primarily as delays in growth and development, and can be reversed by thyroid hormone administration. We are not aware of any evidence showing that human hypothyroidism is associated with chondronecrosis, and given the unremarkable phenotype of D1 and D2 deficient mice [32], it is unlikely that T3 deficiency within osteo-chondroprogenitor cells would, by itself, be able to account for the skeletal phenotype of *Col2a1-Cre*; *Trsp^fl/fl^* mice. Lastly, given that very low serum levels of selenium and iodine almost invariably coexist in individuals within Kashin-Beck disease endemic regions [1], it raises the possibility that our dietary iodine-proficient *Col2a1-Cre*; *Trsp^fl/fl^* mice may not entirely mimic the human syndrome. Indeed, it is conceivable that low iodine levels, and hence reduced thyroid hormone activity, might actually be protective in the presence of profound selenium deficiency.

The topic of iodine and selenium supplementation in individuals residing in Kashin-Beck disease endemic areas and in those exhibiting the clinical manifestations of this disorder has been reviewed by Vanderpas [37]. In general, epidemiological studies have suggested that either selenium or iodine supplementation decrease the incidence and/or clinical severity of Kashin-Beck disease. However, selenium supplementation in established disease appeared not to have a beneficial clinical effect [38]. Regarding this latter finding, it could be argued that once clinical manifestations are present, some degree of permanent damage to joint and epiphyseal cartilage has already occurred. Clearly, selenium and iodine supplementation in Kashin-Beck endemic areas should be instituted during gestation and then continued until skeletal maturity has been reached so as to prevent growth plate and articular cartilage damage, and sequelae such as secondary osteoarthritis.

We have shown that the *Trsp* gene, and hence, preservation of selenoprotein activity in osteo-chondroprogenitors, is essential to murine skeletogenesis and the maintenance of cartilage viability. Indeed, loss of *Trsp* was associated not only with defects in cartilage and bone development, but also severe chondronecrosis of auricular and tracheal cartilages. Our findings lend support to the idea of selenium deficiency being a key factor in the pathogenesis of the skeletal abnormalities of Kashin-Beck disease.

## Materials and Methods

### Generation of mice

Mice with floxed *Trsp* alleles [15], after being backcrossed (N = 6) onto the C57BL/6J genetic background, were then interbred with mice expressing Cre recombinase under the control of a type II collagen (*Col2a1*) gene promoter [19]. The latter were originally of a mixed background when purchased from The Jackson Laboratory (Bar Harbor, ME) but were subsequently fully (N>10) backcrossed onto the C57BL/6J genetic background prior to their use in the experiments reported herein. Tail tip clippings from pups were taken immediately post-weaning and processed for genotyping for both the *Trsp^fl/fl^* and the Cre transgene. Cartilage-specific excision of *Trsp* was verified using a previously PCR reaction [15]. Specific pathogen-free mice were maintained on standard mouse chow (Pico-Vac Lab Mouse Diet #5062, Brentwood, MO), and housed in a barrier facility in accordance with both University of Calgary Animal Care Committee and Canadian Council on Animal Care guidelines.

### Weights and lengths of mice

For identification purposes, newborn pups were marked with dots on the base of the footpad according to the Ketchum Manufacturing's (Brockville, ON, Canada) tattooing protocol. Weights were determined every three to four days for up to three weeks, and length measurements (from the tip of the nose to the base of the tail) were taken at birth and again at 3.5 and 4 weeks of age.

### Microcomputed tomography

Micro-CT scanning (*vivaCT 40*, Scanco Medical, Basserdorf, Switzerland) was performed at different locations on 4 wk old mice (skull, spine, knees). Scanning was done at 10 µm isotropic resolution (55 kVp, 109 µA, 400 ms integration time, 2,000 projections on 360°, 2048 CCD detector array, and cone-beam reconstruction) and images were taken at various orientations and cutplanes. X-ray images of the whole animal or of distinct regions were also taken using this instrument. The generalized shape images were obtained as described [39],[40]. This refers to the mean shape as obtained through scaling, superimposition, and averaging of the volumetric image data for the entire sample. For the cortical analysis, a 1.5 mm section of the mid-shaft of the femur was scanned and evaluated using semi-automatically drawn counters and were thresholded and Gaussian filtered (sigma = 1.2, support = 1), to form binarized images upon which measures of cortical thickness could be obtained [41].

### Histopathology

Knees were fixed in 4% paraformaldehyde for 7 days then decalcified in 14% EDTA. Ear samples were fixed in 10% formalin, and lungs were inflated and fixed with 10% formalin delivered via a cannula inserted into the proximal trachea. Samples from knees, ears and trachea were embedded into 50∶50 paraffin blocks and cut into 4 µm sections. Histology stains included hematoxylin and eosin, and a triple-stain, consisting of hematoxylin, fast green and safranin-O. For detection of proliferating cells, mice were injected with 100 mg/kg BrdU 2 hrs prior to euthanasia; replicating cells were detected via staining with a BrdU Kit (Zymed, Invitrogen Corporation, Carlsbad, CA); alternatively, cell proliferation was assessed using an anti-PCNA antibody (1∶50, Santa-Cruz Biotechnology, Santa Cruz, CA) in combination with the Vectastain ABC Rabbit Kit, (Burlingame, CA), using hematoxylin as the counter-stain. Apoptosis was measured using the TUNEL method (Chemicon Apoptag kit, Millipore, Billerica, MA) with methyl-green counterstaining. Quantification of proliferating and apoptotic cells was done by averaging positive cells from 2–4 serial sections within the proliferative zone of the growth plate of both the distal femurs and proximal tibiae of the mice.

### Immunoblotting

Rabbit antibodies raised against thioredoxin reductase 1 (TR1) were prepared in the laboratory of DLH. Anti-rabbit-HRP-conjugated secondary antibody was obtained from Sigma (St. Louis, MO); NuPAGE 4–12% polyacrylamide gels and PVDF membranes were from Invitrogen (Carlsbad, CA), and SuperSignal West Dura Extended Duration Substrate was from Thermo Scientific (Rockford, IL). Chondrocyte samples were isolated from ribs of 10 day old mice through successive digestions with pronase (2 mg/ml) and collagenase D (3 mg/ml). Chondrocyte and liver protein extracts prepared from control and knockout mice were subjected to electrophoresis on NuPAGE 4–12% polyacrylamide gels, transferred to PVDF membranes and immunoblotted with anti-TR1 antibody (1∶1000 dilution). Membranes were washed with 0.1% TBS-T (20 mM Tris/HCl, pH 7.5, 150 mM NaCl and 0.1% Tween 20) and anti-rabbit-HRP-conjugated secondary antibody (1∶20000) was used. Following incubation with the secondary antibody, membranes were washed with 0.1% TBS-T, incubated in SuperSignal West Dura Extended Duration Substrate and exposed to X-ray film.

### Statistics

Where applicable, samples were compared using a standard student's *t*-test.
